# Gastric bypass surgery in lean adolescent mice prevents diet-induced obesity later in life

**DOI:** 10.1038/s41598-019-44344-7

**Published:** 2019-05-27

**Authors:** Michael B. Mumphrey, Zheng Hao, R. Leigh Townsend, Emily Qualls-Creekmore, Sangho Yu, Thomas A. Lutz, Heike Münzberg, Christopher D. Morrison, Hans-Rudolf Berthoud

**Affiliations:** 1 Neurobiology of Nutrition & Metabolism Department, Pennington Biomedical Research Center, Louisiana State University System, Baton Rouge, LA USA; 2 Institute of Veterinary Physiology, Vetsuisse Faculty, University of Zürich, Zürich, Switzerland

**Keywords:** Gastrointestinal models, Obesity

## Abstract

Gastric bypass surgery is the most effective treatment and is often the only option for subjects with severe obesity. However, investigation of critical molecular mechanisms involved has been hindered by confounding of specific effects of surgery and side effects associated with acute surgical trauma. Here, we dissociate the two components by carrying out surgery in the lean state and testing its effectiveness to prevent diet-induced obesity later in life. Body weight and composition of female mice with RYGB performed at 6 weeks of age were not significantly different from sham-operated and age-matched non-surgical mice at the time of high-fat diet exposure 12 weeks after surgery. These female mice were completely protected from high-fat diet-induced obesity and accompanying metabolic impairments for up to 50 weeks. Similar effects were seen in male mice subjected to RYGB at 5–6 weeks, although growth was slightly inhibited and protection from diet-induced obesity was less complete. The findings confirm that RYGB does not indiscriminately lower body weight but specifically prevents excessive diet-induced obesity and ensuing metabolic impairments. This prevention of obesity model should be crucial for identifying the molecular mechanisms underlying gastric bypass surgery.

## Introduction

The obesity epidemic is increasingly affecting children and adolescents^1,2^, leading to devastating secondary diseases such as type 2 diabetes mellitus, non-alcoholic fatty liver disease and hepatic fibrosis, and cardiovascular disease^3–6^. In addition, the serious psychological problems associated with stigmatization and bullying drastically reduce quality of life and life expectancy^7^. Although coordinated behavioral, dietary, psychological, and medical therapy can be effective in curbing weight gain and disease progression for a while^7–9^, long-term effectiveness is rather disappointing^5^.

Bariatric surgery is increasingly considered in severely obese adolescents and even children and an intensive debate of pros and cons has begun^7,9–16^. All types of bariatric surgeries have been performed in adolescents, and although postsurgical observation periods have been relatively short, the outcomes were comparable in safety and efficacy to surgeries in adults. In one study with a longer follow-up of 7 years after Roux-en-Y gastric bypass (RYGB) in obese adolescents of less than 18 years of age, average BMI dropped from 38.9 to 27.5 kg/m2, all patients resolved their initial comorbidities, and compliance with postoperative guidelines was good in 16 out of 19 patients^17^. A number of other studies with much shorter follow-up periods of 1–3 years generally find all types of bariatric surgeries to be as safe and effective in adolescents as they are in adults^18–22^. Even in children between the ages of 7–14 years, laparoscopic vertical sleeve gastrectomy (n = 116) was safe and resulted in significant weight loss, improved growth and resolution of comorbidities without mortality^23^.

While the outcomes of bariatric surgeries in children and adolescents are generally as impressive as in adults, studies with longer-term follow up are urgently needed. Bariatric surgeries have been modeled in rodents with remarkably similar outcomes, with the advantage that long-term effects and underlying mechanisms are much easier to study. In particular, the mouse model of RYGB surgery has demonstrated sustained weight loss even in the face of continued exposure to high-fat Western diets^24–26^ and a number of transgenic mouse models have been used to test specific potential underlying mechanisms^25,27–31^. However, a weakness of these reversal-of-obesity models has been the difficulty to distinguish between weight loss-dependent from weight loss-independent mechanisms, because surgery-specific effects are superimposed on acute, non-specific effects such as surgical trauma, analgesics, and the severe hypocaloric diet imposed.

The aim of the present study was twofold, first to establish a prevention-of-obesity mouse model, in which surgery is carried out in lean mice followed much later by a high-fat dietary challenge, at a time when the acute surgical effects have long been gone. Second, we wanted to test the feasibility of performing RYGB in young female and male mice that could serve as a model for bariatric surgeries in children and adolescents.

## Materials and Methods

### Animals and diets

Male and female C57/BL6J mice from an in-house breeding colony were weaned and individually housed on regular (low-fat) chow before and for about 12 weeks after RYGB or sham surgery. At about 12 weeks after surgery, a two-choice diet consisting of high-fat (60% energy from fat, Research Diets D12492) and low-fat (13% energy from fat, regular laboratory chow, Purina 5001) was provided. After 35 weeks on the two-choice diet, female mice were additionally exposed to 30% liquid sucrose (99.5% Sucrose, Sigma-Aldrich, St. Louis, MO, dissolved in distilled water) creating a three-choice diet, as free-choice high-fat high-sucrose diets can produce super obesity^32^. Animals were kept in climate-controlled rooms (23–24 °C) and a 12/12 hour light-dark cycle (lights on at 07:00), except for periods of temperature near thermo-neutrality in metabolic chambers.

Animal care and experimentation was approved by the Pennington Biomedical Research Center Institutional Animal Care and Use Committee and strictly followed rules and guidelines provided by the American Physiological Society and NIH.

### Experimental overview

#### Female mice

Adolescent female mice at 5–6 weeks of age and weighing about 16 g were assigned to one of four groups: Roux-en-y gastric bypass surgery (RYGB, n = 8), sham surgery (sham, n = 6), no surgery high-fat diet control (HFD, n = 3), or no surgery chow control (chow, n = 3). Sample size in the latter two control groups was small, as there are plenty of data showing the typical body weight curves of chow fed (https://www.jax.org/-/media/jaxweb/images) and high-fat-fed C57/BL6J mice (Sorhede *et al*., Diabetes 2004). RYGB and sham mice were exposed to the two-choice high-fat diet about 12 weeks after surgery and non-surgical controls were exposed to either the two-choice diet or regular chow at a similar age. At 35 weeks after introduction to the two-choice diet, 30% liquid sucrose was given in addition to the two-choice solid diet, and the study was terminated at about 62 weeks after surgery and 50 weeks after the start of high-energy diet.

#### Male mice

A first cohort of 4.5–5 week-old adolescent male mice weighing about 18 g were assigned to one of two groups: Roux-en-Y gastric bypass surgery (RYGB, n = 5), or sham surgery (sham, n = 5). A second cohort of 5–6 week-old male mice weighing about 22 g was assigned to one of four groups: Roux-en-Y gastric bypass surgery (RYGB, n = 8), sham surgery (sham, n = 8), no surgery high-fat diet control (HFD, n = 4), or no surgery chow control (chow, n = 4).

All mice were singly housed and weighed every 1–5 days. Body composition was measured just before surgery and every 2 weeks thereafter. Food intake was measured from 3 days before to 3 weeks after introduction of the high-fat diet. In the second cohort of males, food intake was again measured for 1 week at 23 weeks after introduction of the high-fat diet. No other invasive tests such as glucose tolerance or metabolic chambers were conducted before exposure to the diet 12 weeks after surgery, so as not to distort growth curves. After the start of high-fat diet, glucose tolerance and insulin tolerance tests were performed at various time points as indicated in the results, and energy expenditure was measured in metabolic chambers at about 6 and 30 weeks after start of high-fat diet. At termination, trunk blood was collected and tissues were weighed and harvested.

### RYGB and sham surgery

RYGB surgery was performed following an established protocol as described in detail earlier^33^. Briefly, the jejunum was transected at about 7–8 cm from the pylorus and the stomach was transected near the esophagus. The lower end of the cut jejunum was anastomosed to the small gastric pouch and the upper end of the cut jejunum was anastomosed to the side of the lower jejunum. This resulted in Roux-, biliopancreatic-, and common-limbs of approximately 5, 8, and 21 cm in females, and 6, 10, and 26 cm in males, respectively (as measured post mortem). Sham surgery consisted of anesthesia, laparotomy and re-closure.

### Measurement of food intake and food choice

Food intake in kcal/day was assessed by weighing daily amounts of chow (3.02 kcal/g) and high-fat diet (5.24 kcal/g) eaten and taking spillage into account. Sucrose intake (30% sucrose = 1.35 kcal/g) was measured by weighing drinking bottles daily. Preference for each diet was calculated as the average daily kcal consumed for that diet as a percent of total daily kcal consumed.

Because measurement of food intake requiring grid floors is stressful to mice and can influence body weight, it was only measured during short critical periods of the experiment, including just before and after introduction of the high-fat diet and just before termination of the experiment.

### Measurement of body composition

Body composition was measured just before high-fat exposure and every 2–4 weeks thereafter without anesthesia using a Minispec LF90 NMR Analyzer (Bruker Corporation, The Woodlands, TX). Adiposity index was defined as fat mass divided by lean mass.

### Measurement of energy expenditure, RER, and locomotor activity

VO_2_, VCO_2_, respiratory exchange ratio (RER), and locomotor activity were measured once food intake stabilized after exposure to the high-fat diet (43 ± 5 days, referred to as 6 weeks, in females; 40 ± 9 days, referred to as 6 weeks, in males), and once more towards the end of the study (220 ± 14 days, referred to as 31 weeks, in females; 204 ± 25 days, referred to as 29 weeks, in males) in metabolic chambers (Phenomaster/Lab Master, TSE Systems, Germany). All mice were first adapted to eating food from hanging baskets in training cages for 4–10 days, and mice that had difficulty eating from hanging baskets were floor-fed throughout the training and measurement period. For all measurement periods, mice were first kept at room temperature (23 °C) for 3 days, and then at thermoneutrality (29 °C) for 3–4 days. Mice were allowed to adapt to each condition for at least one day before taking measurements.

All measurements were calculated as an average per 24 hours, with a 24 hour period including 12 hours in the dark and 12 hours in the light. Energy expenditure was calculated on the basis of VO_2_ and VCO_2_ by company supplied software, and was adjusted to total body mass by ANCOVA. Locomotor activity was defined as the total number of laser beam breaks in the x and y planes (7 mm spatial resolution, 10 ms temporal resolution) over the given time period.

### Glucose and insulin tolerance tests

Glucose tolerance tests (GTT) were performed a few weeks after the first period in the metabolic chambers (time after exposure to high-fat diet: 93 ± 4 days, referred to as 13 weeks, in females; 89 ± 6 days, referred to as 13 weeks, in males). Each GTT took place between 09:00–12:00 h after 3–5 hours of food deprivation. Mice were given an i.p. injection of α-D-glucose at a dose of 1.5 g/kg body weight, and blood glucose was measured before and at 15, 30, 60, and 120 minutes after injection by taking a small sample of blood from the tail vein and assessing it with glucose strips and a glucometer (Onetouch Ultra Strips and Onetouch Ultra Glucometer, LifeScan INC, Milpitas, CA), which can read blood glucose concentrations up to 600 mg/dL.

Insulin tolerance tests (ITT) were performed just before the second period in the metabolic chambers (time after exposure to high-fat diet: 201 ± 1 days, referred to as 29 weeks, in females; 174 ± 27 days, referred to as 25 weeks, in males). Each ITT took place between 09:00–12:00 h after 3–5 hours of food deprivation. Mice were given an i.p. injection of insulin (Novolin R, Novo Nordisk Inc., Plainsboro, NJ) at a dose of 0.6U/kg body weight, and blood glucose was measured with the same methods used for the GTT.

### Fasting insulin, leptin, glucose, and active GLP-1

Fasting plasma insulin (3–5 hours food deprived) was measured using a blood sample taken just before the first GTT, and again at termination of the experiment. For the first measurement, a 50 µl blood sample was taken from the tail vein using a heparinized capillary tube (Fisherbrand Microhematocrit Capillary Tubes, Thermo Fisher Scientific, Waltham, MA), and for the second measurement a 500 µl sample of trunk blood was added directly to 83.5 µl EDTA. In both cases, a protease inhibitor cocktail was immediately added to the sample (equal parts of each of the following: Protease inhibitor, Sigma-Aldrich, St. Louis, MO; DPP-IV inhibitor, EMD Millipore, St. Charles, MO; Pefabloc SC, Roche, Indianapolis, IN) at a ratio of 15 µl protease inhibitor cocktail to 500 µl blood. The sample was centrifuged at 4 °C and 3000 RPM for 10 minutes, and plasma was separated from the whole blood. Plasma aliquots were frozen in liquid nitrogen and stored at −80 °C until processing. Plasma was subjected to ELISA for insulin concentrations (MADKMAG-71k, MILLIPLEX MAP Mouse Adipokine Magnetic Bead Panel, EMD Millipore, St. Charles, MO). Fasting plasma leptin was measured only at termination of the experiment from the same trunk blood sample, using the same methods as for fasting plasma insulin.

Homeostatic model assessment of insulin resistance (HOMA-IR) at 13 weeks was calculated using the baseline measurement of blood glucose obtained during the GTT and the plasma insulin assay results. HOMA-IR at the termination of the experiment was calculated using blood glucose measured from a fresh drop of trunk blood that had not come into contact with EDTA or protease inhibitors and the same glucometer used in the GTT and ITT, and the terminal insulin assay results. HOMA-IR was calculated as Glucose (mg/dL) x Insulin (mU/L)/405.

Active GLP-1 in plasma of 3–5 h food deprived female and male mice was measured in duplicate with ELISA (EZGLP1T-36K assay, Millipore Sigma, Burlington, MA).

### Liver weight and lipid content

At termination of the study, the liver was weighed and a lobe from the liver was removed, immediately placed in 10% neutral buffered formalin for fixation, and stored at 4 °C until processing. Fixed liver samples were rinsed in PBS and then soaked overnight in a 30% sucrose solution. Samples were then embedded in OCT (Tissue-Tek O.C.T. Compound, Sakura Finetek USA, Torrance, CA), snap frozen, and sectioned on a cryostat at a thickness of 8 µm. Frozen sections were immediately placed on poly-L-lysine coated glass slides (Poly-Prep Slides, Sigma-Aldrich, St. Louis, MO) and allowed to dry for at least an hour at room temperature.

Sections were stained with oil red O (oil red O solution, Sigma-Aldrich, St. Louis, MO) and counterstained with Mayer’s hematoxylin (Hematoxylin Solution, Mayer’s, Sigma-Aldrich, St. Louis, MO). Slides with dried sections adhered were dipped two times in distilled water for 5 seconds, dipped two times in 60% isopropanol for 5 seconds, and then were submerged in the oil red O solution and incubated covered for 15 minutes. Afterwards slides were again dipped twice in 60% isopropanol for 5 seconds, then twice in distilled water for 5 seconds, and then incubated in Mayer’s Hematoxylin (Sigma-Aldrich, St. Louis, MO) for 60 seconds. Slides were then gently rinsed under tap water for 3 minutes and immediately cover slipped using an aqueous mounting medium (Fluoromount-G, Thermo Fisher Scientific, Waltham, MA). Slides were digitally scanned using a NanoZoomer 2.0-HT Whole Slide Imager (Hamamatsu Photonics, Hamamatsu City, Shuzuoka, Japan), and pictures were generated from digital scans using company supplied software.

### Bone density

Femurs were dissected at the end of the experiment and bone mineral density (BMD) was assessed on isolated femurs with micro computed tomography^34,35^. Peripheral quantitative computed tomography (XCT 960 A Knochen Scanner; Stratec Medizinaltechnik, Pforzheim, Germany) of the femurs was performed in the middle of the diaphysis and at the level of the epiphysis (trabecular bone), and BMD was determined.

### Statistical analysis

Statistical analysis was performed using RStudio version 1.0.153 (RStudio, Inc, Boston, MA). Statistical significance was determined by two-factor ANOVA or repeated measures ANOVA followed by either unpaired *t* tests or pairwise *t* tests with Benjamini-Hochberg corrections (FDR = 0.05). Results were considered significant at p < 0.05. Data in line graphs are presented as mean ± SEM. Data in dotplots are presented as individual data points overlaid on a box showing mean ± SEM. Energy expenditure was additionally analyzed using a one-way analysis of covariance (ANCOVA) to determine the significance of changes in daily energy expenditure after controlling for the confounding effects of body weight. ANCOVA was conducted using the general linear model procedure within the SAS software package (SAS V9, SAS Institute, Cary, NC), with body weight as the covariate. BW-adjusted means and post-hoc comparisons were made using the LSMEANS statement with the PDIFF option, representing least significant differences tests for pre-planned comparisons. Results were considered significant at p < 0.05.

## Results

### RYGB does not significantly affect growth of 5 week-old female mice

**Figure 1:**
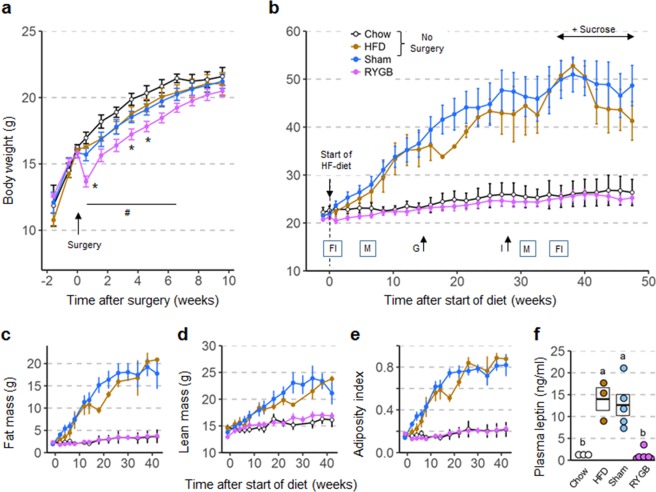
Body weight (**a**,**b**), body composition (**c**–**e**), and plasma leptin levels (**f**) of female mice subjected to RYGB at 5 weeks and exposed to high-fat diet at ~17 weeks of age. (**a**) Body weight curves of mice subjected to RYGB (purple, n = 8), Sham surgery (blue, n = 7), or no surgery (brown and open circles, n = 6) at 5 weeks of age. Note normal growth of mice with RYGB with no significant difference in body weight compared to all other groups at 7 weeks after surgery. *p < 0.05, RYGB vs. Sham; ^#^p < 0.05, RYGB vs. no surgery. (**b**) Body weight of mice with prior RYGB (purple, n = 8), Sham surgery (blue, n = 7), no surgery subjected to high-fat diet (brown, n = 3), or no surgery subjected to chow diet (open circles, n = 3) for 48 weeks. Note complete resistance to high-fat diet-induced obesity in mice with RYGB. Times of measurements of food intake (FI), energy expenditure and activity in metabolic chambers (M), glucose tolerance (G), and insulin tolerance (I) are indicated above the x-axis. (**c**–**e**) Fat mass, lean mass, and adiposity index measured before and after exposure to high-fat diet. (**f**) Plasma leptin levels measured at 13 weeks after exposure to high-fat diet. Data are shown as mean ± SEM, or individual data points overlaid on a box showing mean ± SEM. Groups that do not share the same letters are significantly different from each other (p < 0.05, pairwise t-tests with Benjamini-Hochberg correction, FDR = 0.05).

Although RYGB induced initial weight loss, body weight was no longer significantly different from sham-operated female mice and age-matched mice without surgery at 7–10 weeks after RYGB (Fig. 1a). At the time of introduction of the two-choice high-fat diet ~12 weeks after surgery, average body weight (Fig. 1b) fat mass (Fig. 1c), lean mass (Fig. 1d), and adiposity index (Fig. 1e) were not significantly different between all 4 groups. Importantly, body weight 10 weeks after surgery (~16 weeks of age) of all groups were similar to the body weight of undisturbed, group-housed C57/BL6J mice as published by Jackson Labs (https://www.jax.org/-/media/jaxweb/images). Also, there was no mortality or complications in any of the mice with RYGB surgery.

### RYGB reduces growth in 5 week-old but not in 6–7 week-old male mice

**Figure 2:**
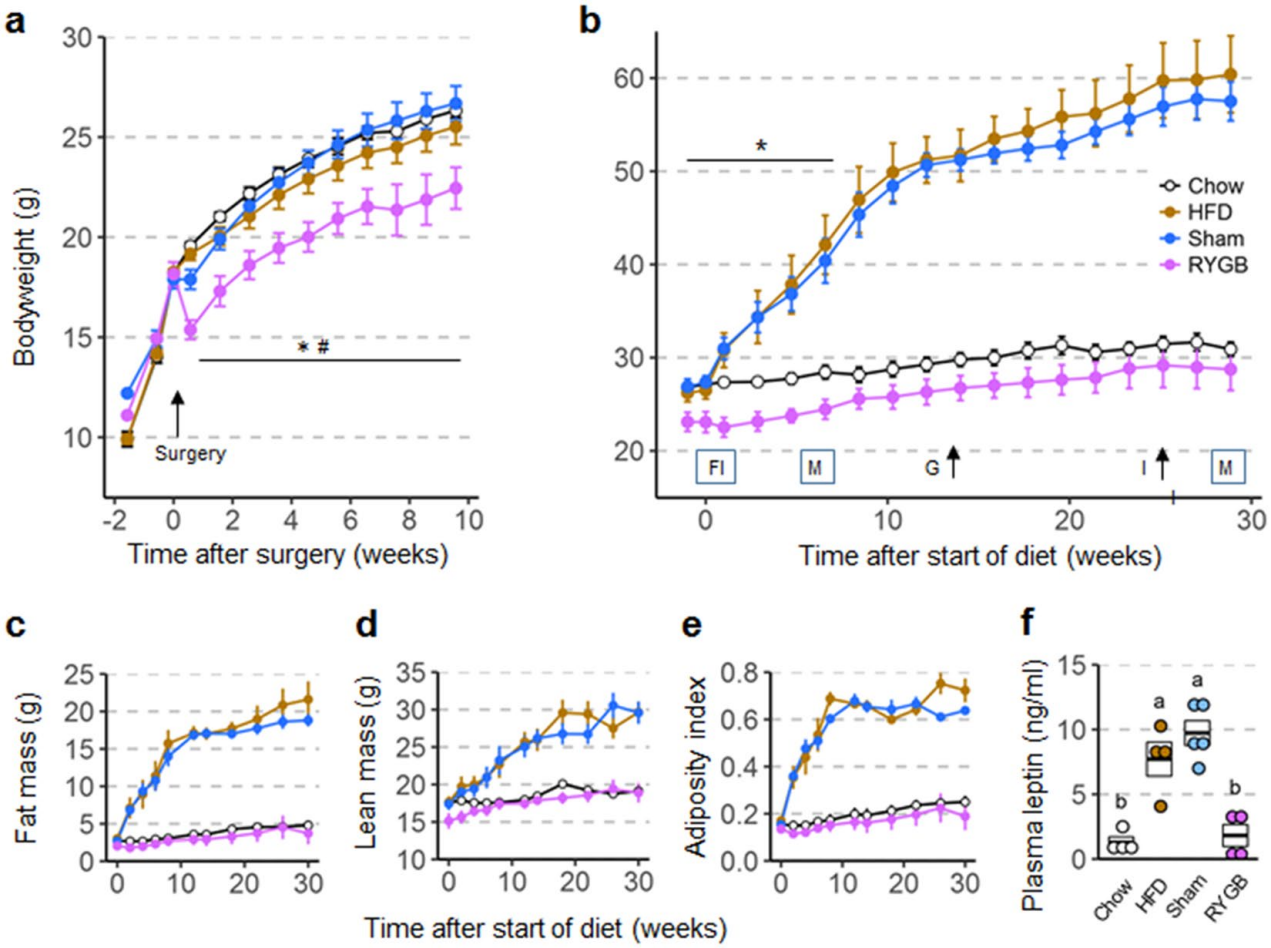
Body weight (**a**,**b**), body composition (**c**–**e**), and plasma leptin levels (**f**) of male mice subjected to RYGB at 5 weeks and exposed to high-fat diet at ~17 weeks of age. (**a**) Body weight curves of mice subjected to RYGB (purple, n = 5), Sham surgery (blue, n = 5), or no surgery (brown and open circles, n = 8) at 5 weeks of age. Note reduced growth of mice with RYGB with significant difference in body weight at 10 weeks after surgery compared to all other groups. *p < 0.05, RYGB vs. Sham; ^#^p < 0.05, RYGB vs. no surgery. (**b**). Body weight of mice with prior RYGB (purple, n = 5), Sham surgery (blue, n = 5), no surgery subjected to high-fat diet (brown, n = 4), or no surgery subjected to chow diet (open circles, n = 4) for 29 weeks. Note complete resistance to high-fat diet-induced obesity in mice with RYGB. Times of measurements of food intake (FI), energy expenditure and activity in metabolic chambers (M), glucose tolerance (G), and insulin tolerance (I) are indicated above the x-axis. (*p < 0.05, RYGB vs Chow) (**c**–**e**). Fat mass, lean mass, and adiposity index measured before and after exposure to high-fat diet. (**f**) Plasma leptin levels measured at 13 weeks after exposure to high-fat diet. Data are shown as mean ± SEM, or individual data points overlaid on a box showing mean ± SEM. Groups that do not share the same letters are significantly different from each other (p < 0.05, pairwise t-tests with Benjamini-Hochberg correction, FDR = 0.05).

Five week-old male mice weighing ~18 g at the time of surgery showed the same transient weight loss followed by near normal growth as did their female counterparts. However, they did not quite catch up with sham-operated mice and age-matched mice without surgery (Fig. 2a), so that at the time of introduction of the high-fat diet at 11–13 weeks after surgery, they weighed significantly less than mice with sham surgery (−16%, p < 0.05) and mice without surgery (−13% vs. HFD, p < 0.05; −15% vs. Chow, p < 0.05) (Fig. 2b). Although there was a slight reduction in fat mass, lean mass, and adiposity index in mice with RYGB when compared to mice with sham and mice without surgery before introduction of the high-fat diet, none of these differences reached significance (Fig. 2c–e).

**Figure 3:**
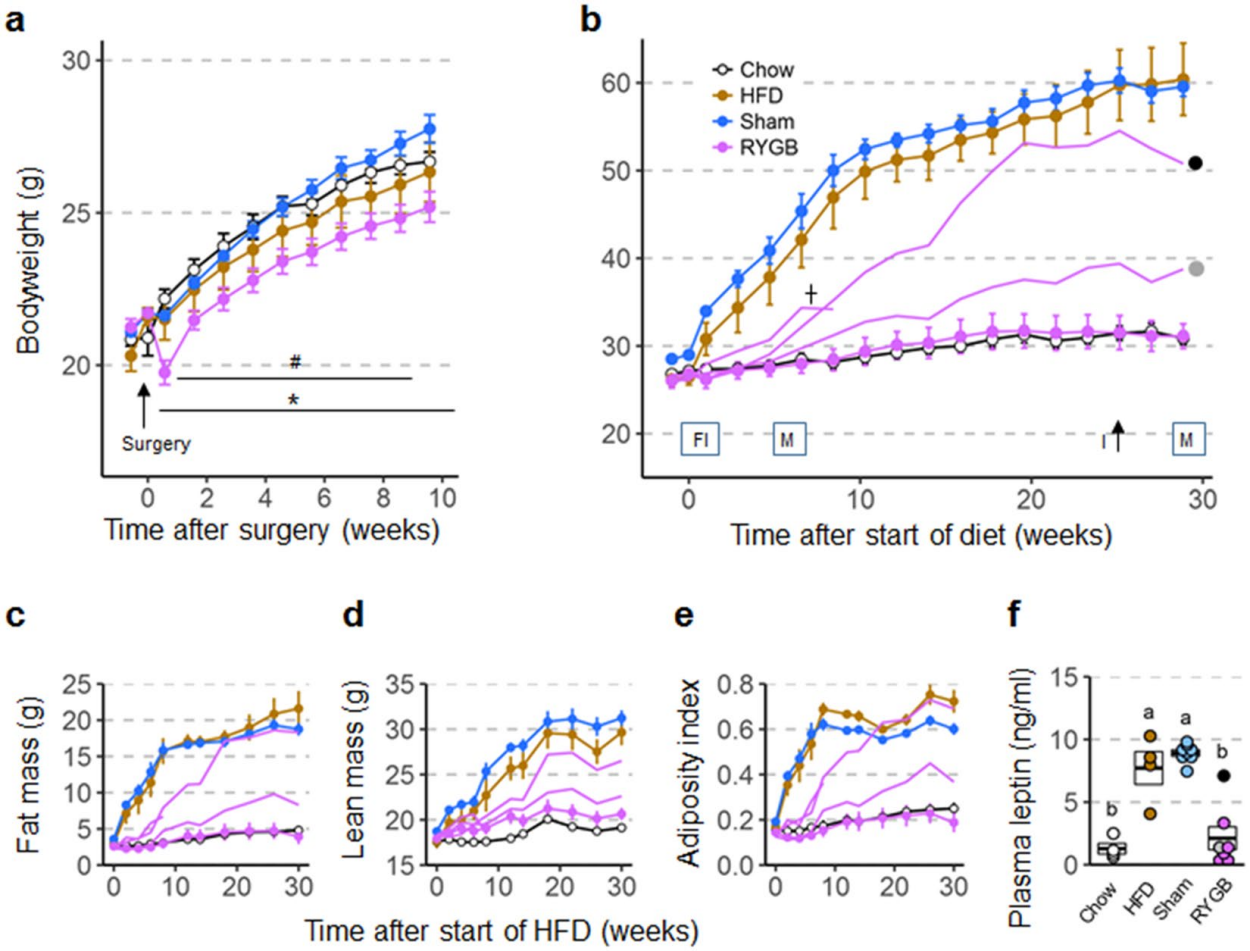
Body weight (**a**,**b**), body composition (**c**–**e**), and plasma leptin levels (**f**) of male mice subjected to RYGB at 6–7 weeks and exposed to high-fat diet at ~18 weeks of age. (**a**) Body weight curves of mice subjected to RYGB (purple, n = 8), Sham surgery (blue, n = 8), or no surgery (brown and open circles, n = 8) at 6 weeks of age. *p < 0.05, RYGB vs. Sham; ^#^p < 0.05, RYGB vs. no surgery. (**b**) Body weight of mice with prior RYGB (DIO resistant, n = 5; not DIO resistant, n = 3), Sham surgery (blue, n = 8), no surgery subjected to high-fat diet (brown, n = 4), or no surgery subjected to chow diet (open circles, n = 4) for 29 weeks. Note complete resistance to high-fat diet-induced obesity in 5 mice with RYGB, but various degrees of obesity in 3 outliers (shown by the 3 separate purple lines and the gray and black dot). Times of measurements of food intake (FI), energy expenditure and activity in metabolic chambers (M), glucose tolerance (G), and insulin tolerance (I) are indicated above the x-axis. (**c**–**e**) Fat mass, lean mass, and adiposity index measured before and after exposure to high-fat diet. (**f**) Plasma leptin levels measured at 13 weeks after exposure to high-fat diet. Data are shown as mean ± SEM, or individual data points overlaid on a box showing mean ± SEM. Groups that do not share the same letters are significantly different from each other (p < 0.05, pairwise t-tests with Benjamini-Hochberg correction, FDR = 0.05).

To investigate whether surgery at a slightly older age would retard body weight gain less, a second cohort of 6–7 week old male adolescent mice weighing ~22 g was subjected to surgery. In this cohort, body weight was less inhibited by RYGB (Fig. 3a). At the time of high-fat diet exposure, the body weight of RYGB mice was slightly lower than the sham mice (−8%, p < 0.05) (Fig. 3b), but there was no difference between RYGB and the non-surgical controls. The slightly lower body weight of RYGB compared with sham mice was mainly due to lower fat mass (−30%, p < 0.01) and not lean mass (−4%, p > 0.05), resulting in a significantly lower adiposity index (−28%, p < 0.01) at the time of exposure to high fat diet (Fig. 3c–e). Again, there was no mortality or complications in both cohorts with RYGB surgery.

### RYGB completely prevents high-fat diet-induced obesity in female mice

As expected, female mice without surgery or with sham surgery responded promptly to exposure of the two-choice high-fat diet by rapidly gaining body weight and fat mass (Fig. 1b,c). By 30 weeks after the start of high-fat diet these mice more than doubled their body weight, mostly by accruing fat mass as indicated by a four-fold increase in the adiposity index (Fig. 1c,e). Three of the mice with sham surgery developed small skin lesions (likely due to excessive scratching) and were euthanized after a period of weight loss at 25, 38, and 48 weeks after the start of the high-fat diet, respectively, and they were not available for tissue harvest.

In stark contrast, all 8 female mice with RYGB completely resisted weight gain on the high-fat diet up to 50 weeks. At 40 weeks, their body weight was not significantly different from chow control mice, but significantly less than sham and diet controls (Fig. 1b). Total body fat was not significantly different throughout the observation period and fat pad weights were not significantly different at the end of the study compared with chow controls (Supplementary Fig. S1). The absence of obesity in mice with RYGB early in life and exposure to high-fat diet for 50 weeks was also indicated by the normal plasma leptin levels (Fig. 1f). Finally, total gut weight was significantly higher in female mice with RYGB compared with all other groups (Supplementary Fig. S2).

### RYGB incompletely prevents high-fat diet-induced obesity in male mice

In male mice, the general outcome was quite similar to the females. Male sham-operated mice of both cohorts (~18 or ~22 g at surgery) and diet controls exposed to the two-choice high-fat diet initially showed more rapid body weight gain compared to their female counterparts that moderated after about 12 weeks of exposure (Figs 2b, 3b). After 30 weeks on the diet these mice weighed close to 60 g. In cohort 1 (18 g), all 5 male RYGB mice were completely protected from diet-induced obesity, as their body weight, fat mass, and adiposity index at 30 weeks was not significantly different from chow controls (Fig. 2b,c,e). Plasma leptin levels were also not significantly higher in RYGB compared to non-surgical chow controls (Fig. 2f).

In cohort 2 (22 g), 3 of the 8 mice with RYGB developed some degree of obesity. Remarkably, one mouse was initially protected from the diet up to 4 weeks, but then rapidly gained weight to join the obese sham and diet controls by ~16 weeks (black dot in Fig. 3b). Another mouse with RYGB only slowly developed obesity over the 31 week observation period, eventually gaining 52% body weight (gray dot in Fig. 3b). A third mouse with verified RYGB gained 10% body weight in the first week and 40% by 8 weeks of diet exposure, when it suddenly lost weight and was euthanized, with autopsy indicating necrosis of the ileum. The remaining 5 mice with RYGB completely resisted diet-induced obesity, as their body weight, fat mass, adiposity index after 30 weeks, and plasma leptin levels after 13 weeks were not significantly different compared to the non-surgical chow controls. As in females, individual fat pad weights in male mice with RYGB were not significantly different from chow fed controls (Supplementary Fig. S1), but total gut weight was significantly increased (Supplementary Fig. S2).

### Protective effects of RYGB are due to effects on both food intake and energy expenditure

#### Food intake and food choice

**Figure 4:**
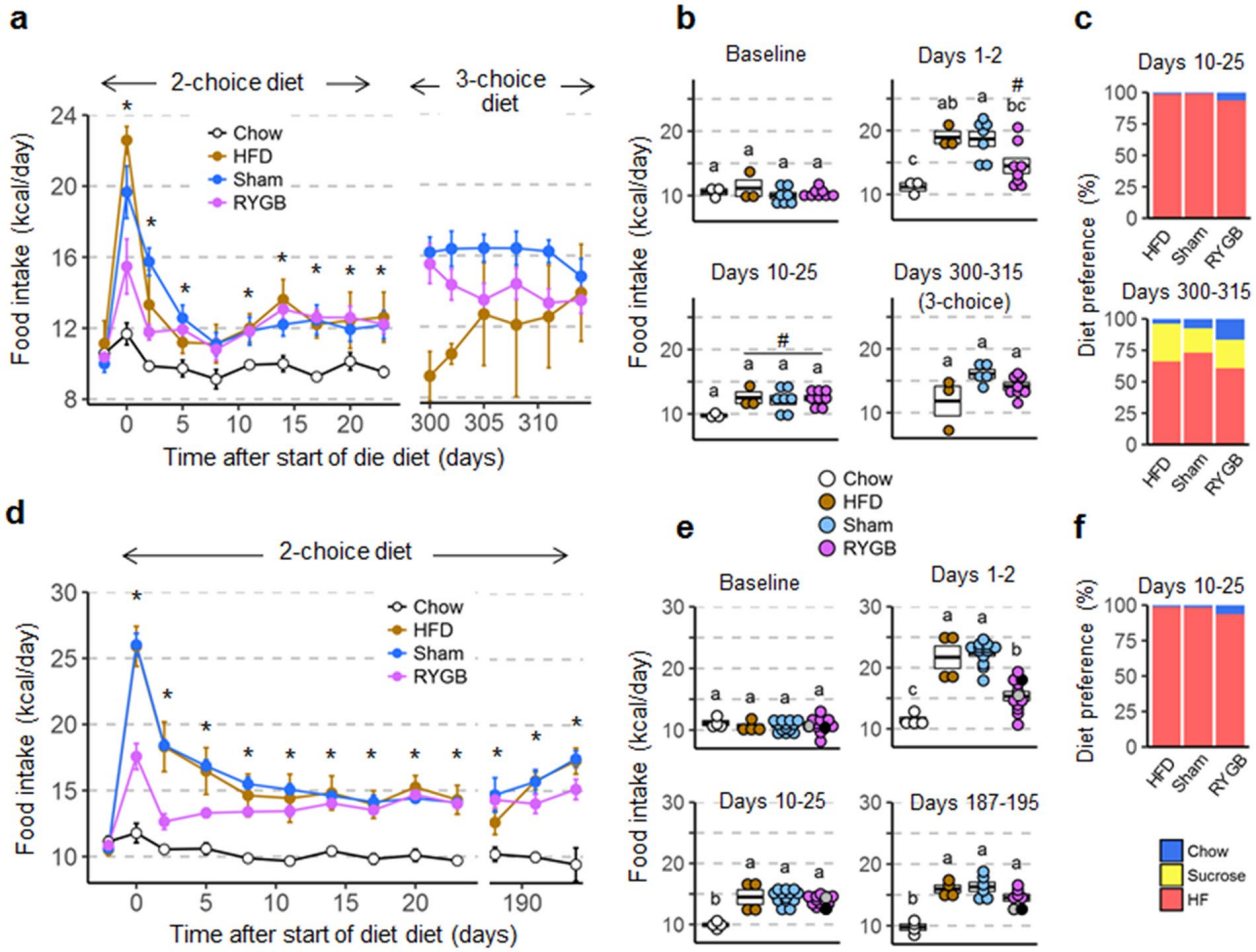
Food intake and food choice of female (**a**–**c**) and male (**d**–**f**) mice subjected to RYGB early and exposed to high-fat diet later in life. (**a**,**d**) Daily food intake before and after exposure to two-choice diet of mice with prior RYGB (purple, Female, n = 8; Male, n = 7–13), sham surgery (blue, Female, n = 5–7; Male, n = 6–13), no surgery subjected to two-choice diet (brown, Female, n = 3; Male, n = 4), or no surgery subjected to chow diet (open circles, Female, n = 3; Male, n = 4). For the female group, food intake at days 300–315 is during exposure to a three choice diet (high fat, sucrose, and chow). Note that food intake data for male mice with RYGB surgery performed at both 5 and 6 weeks were pooled after determining that there were no significant differences between them. *p < 0.05, RYGB vs. chow. (**b**,**e**) Average daily food intake at baseline, immediately after exposure to high-fat diet (Days 1–2), after food intake has stabilized (Days 10–25), and just before termination (Female, days 300–315; Male, days 187–195). The two male mice with RYGB not resisting weight gain are indicated by the gray and black dots. Groups that do not share the same letters are significantly different from each other (p < 0.05, pairwise t-tests with Benjamini-Hochberg correction, FDR = 0.05), # p < 0.06 vs Chow. (**c**) Average diet preference in female mice at 10–25 and 300–315 days after first exposure to high-fat diet. (**f**) Average diet preference in male mice at 10–25 days after first exposure to high-fat diet. Data are shown as mean ± SEM, or individual data points overlaid on a box showing mean ± SEM.

It is important to note that because surgery was performed at a much earlier time point, food intake measurements at the time of high-fat diet exposure were completely free of confounding acute effects of surgery. Baseline chow intake just before the switch to high-fat diet was not significantly different for any groups in both females (Fig. 4a,b) and males (Fig. 4d,e). The strong initial hyperphagic response to high-fat diet of sham-operated and non-surgical mice lasting 1–2 days was significantly reduced, but the sustained, more moderate, hyperphagia during the first 25 days was similar in both female and male mice with prior RYGB. Both male and female mice with prior RYGB consumed about 25–30% more total calories compared to chow controls between days 10–25 after the start of the two-choice high-fat diet exposure. Although this increase did not quite reach statistical significance for the females (p = 0.06, Fig. 4b), it is clear that normal chow intake of female C57/BL6J mice has been reported to be ~10Kal/day, which is considerably lower than the ~12 Kcal in the RYGB group.

The 2-choice chow and high-fat diet allowed measurement of food preference. As we have seen in several previous RYGB-induced reversal-of-obesity studies in male mice and rats^24,36^, both male and female mice with previous RYGB chose a slightly larger percentage of their total intake from chow compared with sham-operated mice (Fig. 4c,f). In addition, some mice of the female cohort were exposed to a three-choice diet, with 30% liquid sucrose in addition to high-fat and chow pellets. Intake of calories from sucrose and sucrose preference were not significantly different for mice with prior RYGB or sham surgery, but variability was high.

#### Energy expenditure

**Figure 5:**
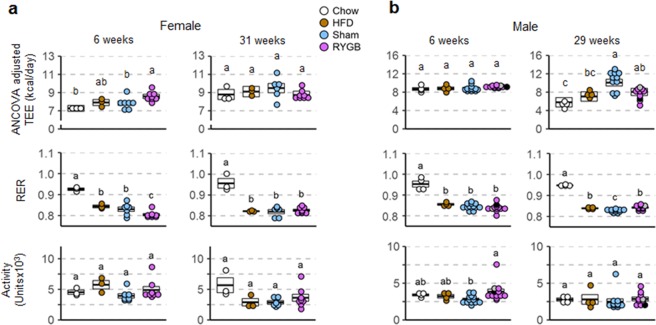
Daily averages of energy expenditure, RER, and activity of female (**a**) and male (**b**) mice assessed at two time points after RYGB surgery at thermoneutrality (29° C). Metabolic parameters of mice with prior RYGB (purple, Female, n = 8; Male, n = 11–13), sham surgery (blue, female, n = 6–7; male, n = 11–13), no surgery subjected to two-choice diet (brown, female, n = 3; male, n = 4), or no surgery subjected to chow diet (open circles, female, n = 3; male, n = 4), after adaptation, were assessed for 4 days in metabolic chambers, 2 days at 23 °C (see Supplementary Fig. S3) and 2 days at 29 °C. Note that all data for male mice with RYGB surgery performed at both 5 and 6 weeks were pooled, after determining that there were no significant differences between them. The two male mice with RYGB not resisting weight gain are indicated by the gray and black dots. Data are individual data points overlaid on a box showing mean ± SEM. Groups that do not share the same letters are significantly different from each other (p < 0.05, pairwise t-tests with Benjamini-Hochberg correction, FDR = 0.05).

Energy expenditure, RER, and locomotor activity were assessed in metabolic chambers at two time points after introduction of the obesogenic diet. Note that to avoid unintended weight loss, we intentionally did not expose the mice to the metabolic chambers or any other invasive treatments before exposure to the diet. Also, because the high baseline energy expenditure of mice kept at the typical room temperature (23 °C) makes it more difficult to detect superimposed changes, we made additional measurements near thermoneutrality (29 °C). Average energy expenditure measured over 2–3 days was generally higher in mice with previous RYGB on high-fat diet compared with chow-fed control mice (with similar body weight and body composition), but the effect varied over time in a sex-specific manner. In female mice with prior RYGB (completely resisting diet-induced obesity), energy expenditure measured near thermoneutrality was significantly higher (+18%, p < 0.05) compared to chow controls at 6 weeks after high-fat diet introduction when adjusted by ANCOVA using total body mass as covariate (Fig. 5a, Supplementary Table 1). In female mice with prior sham surgery or without surgery, both developing obesity, ANCOVA-adjusted energy expenditure was in the middle between chow-fed controls and mice with prior RYGB at 6 weeks (Fig. 5a). However, at 31 weeks after introduction of the high-fat diet, there were no longer any significant differences in energy expenditure, no matter how it was adjusted.

In male mice with prior RYGB (both cohorts were combined after finding no significant differences between them) the reverse time-dependent effects were observed. At 6 weeks after introduction of the high-fat diet there were no significant differences in ANCOVA-adjusted energy expenditure between the 4 groups at thermoneutrality (Fig. 5b, Supplementary Table 1). However, at 29 weeks, ANCOVA-adjusted energy expenditure was significantly and substantially higher (+39%, p < 0.05) compared to chow controls. In male mice with prior sham surgery and developing morbid obesity, ANCOVA-adjusted energy expenditure was slightly but not significantly higher compared to mice with prior RYGB (Fig. 5b).

Although similar trends in energy expenditure were seen at room temperature (23° C), only the increased ANCOVA-adjusted energy expenditure at 6 weeks in female mice with prior RYGB compared to chow was significant, but not the increase at 29 weeks in males (Supplementary Fig. S3, Supplementary Table 1).

There were only small differences in substrate utilization as indicated by the RER between the groups consuming high-fat diet for both sexes (Fig. 5a,b). As expected, only chow-fed controls had a significantly and substantially higher RER compared to all high-fat fed mice.

Locomotor activity assessed in the metabolic chambers at thermoneutrality was not significantly different between RYGB and chow controls for the critical time points when differences in energy expenditure occurred in either female or male mice and is, therefore, unlikely to account for the difference in energy expenditure (Fig. 5a,b).

### RYGB prevents impairments of glycemic control in both females and males

**Figure 6:**
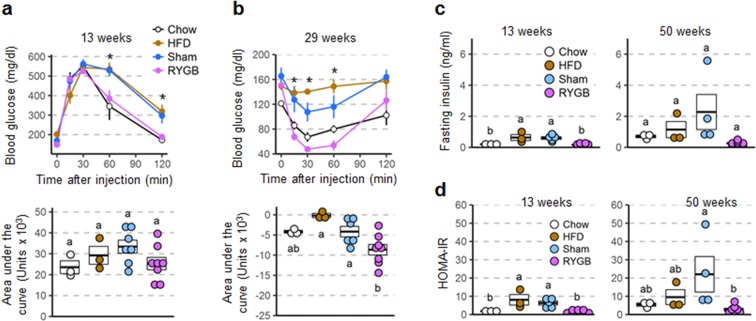
Glycemic control parameters of female mice with RYGB assessed at different time points after exposure to high-fat diet. Glucose tolerance (**a**), insulin tolerance (**b**), as well as fasting insulin (**c**), and HOMA-insulin resistance (**d**) of mice with prior RYGB (purple, n = 8), Sham surgery (blue, n = 4–7), no surgery subjected to high-fat diet (brown, n = 3), or no surgery subjected to chow diet (open circles, n = 3). *p < 0.05 RYGB vs Sham and HFD. Data are shown as mean ± SEM, or individual data points overlaid on a box showing mean ± SEM. Groups that do not share the same letters are significantly different from each other (p < 0.05, pairwise t-tests with Benjamini-Hochberg correction, FDR = 0.05).

In female mice with prior RYGB there was no impairment in glucose tolerance at 13 weeks after exposure to high-fat diet when compared to chow fed controls (Fig. 6a). When compared to sham and non-surgical high-fat fed controls, RYGB mice showed significantly reduced blood glucose levels at 60 and 120 minutes after glucose injection. Total area under the curve for RYGB mice following the GTT was also decreased compared to sham and non-surgical controls, but this trend did not reach statistical significance. Insulin sensitivity was also protected in mice with prior RYGB when compared to both obese controls as measured by insulin tolerance at 29 weeks (Fig. 6b), and fasting plasma insulin levels (Fig. 6c) and HOMA-IR (Fig. 6d) measured at 13 and 50 weeks. However, fasting plasma insulin was not significantly different between RYGB and sham-operated mice (as well as non-surgical chow controls) at the time of sacrifice due to high variability and low sample sizes (Fig. 6c).

**Figure 7:**
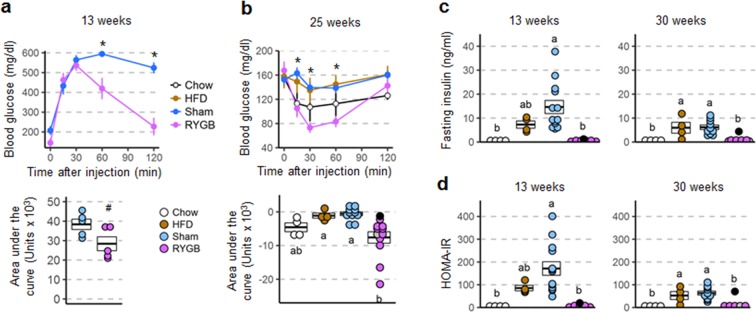
Glycemic control parameters of male mice with RYGB assessed at different time points after exposure to high-fat diet. Glucose tolerance (**a**), insulin tolerance (**b**), as well as fasting insulin (**c**), and HOMA-insulin resistance (**d**) of mice with prior RYGB (purple, n = 5–12), sham surgery (blue, n = 10–13), no surgery subjected to two-choice diet (brown, n = 4), or no surgery subjected to chow diet (open circles, n = 4). Note that all data for male mice with RYGB surgery performed at both 5 and 6 weeks were pooled, after determining that there were no significant differences between them. *p < 0.05 RYGB vs Sham. ^#^p < 0.06 RYGB vs Sham. The two male mice with RYGB not resisting weight gain are indicated by the gray and black dots. Data are shown as mean ± SEM, or individual data points overlaid on a box showing mean ± SEM. Groups that do not share the same letters are significantly different from each other (p < 0.05, pairwise t-tests with Benjamini-Hochberg correction, FDR = 0.05).

In males, the overall effect was similar although the mice in cohort 2 which gained significant body weight on high-fat diet were only partially protected (see next paragraph). Because we did not find significant differences between the two cohorts, data were pooled, except for the first glucose tolerance test at 13 weeks, which was only carried out in cohort 1. Mice with prior RYGB showed significantly lower blood glucose at 60 and 120 minutes after glucose injection when compared to sham controls, and a trend towards a lower area under the curve that did not reach statistical significance (Fig. 7a). Insulin sensitivity was also protected in mice with prior RYGB when compared to sham as measured by insulin tolerance at 25 weeks (Fig. 7b), and fasting plasma insulin levels (Fig. 7c) and HOMA-IR (Fig. 7d) measured at 13 and 30 weeks.

Inspecting glycemic parameters of the 2 male mice with RYGB that were not, or only partially, protected from weight gain upon high-fat exposure shows elevated fasting glucose and impaired glucose tolerance as early as 13 weeks, when body weight had increased by 26 and 54% respectively in the 2 mice (Supplementary Fig. S4).

Fasting active plasma GLP-1 was significantly higher in both female and male mice with prior RYGB, compared with sham surgery (Supplementary Fig. S5).

### RYGB prevents hepatic steatosis in both females and males

**Figure 8:**
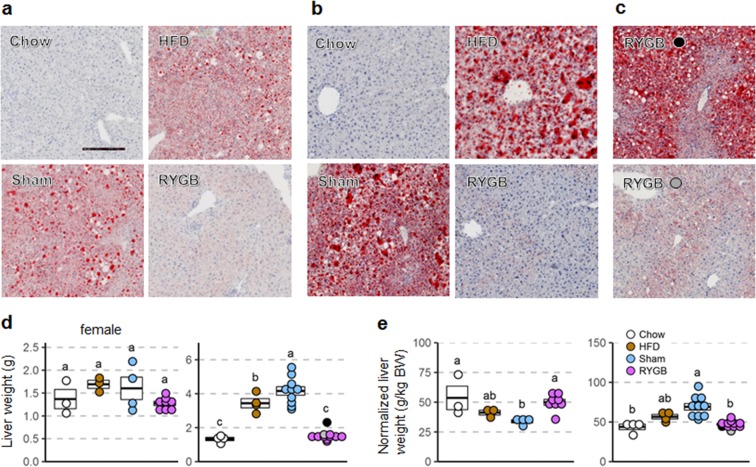
Liver fat and liver weight of female and male mice with RYGB exposed to high-fat diet. A: Representative images of liver fat content (visualized using oil-red-O on a background of hematoxylin staining) of female (**a**) and male (**b**) mice without surgery subjected to regular chow diet (Chow), mice without surgery subjected to high-fat diet (HFD), sham-surgery (Sham), or RYGB. High-fat diet exposure lasted for 30 weeks in females and 50 weeks in males. (**c**) Images for the two mice that regained all (black dot) or substantial (gray dot) body weight after RYGB. (**d**,**e**) Absolute (**d**) and relative (**e**) liver weight of mice with RYGB (purple, female, n = 8; male, n = 11), Sham surgery (blue, female, n = 7; male, n = 11), no surgery subjected to high-fat diet (brown, female, n = 3; male, n = 4), or no surgery subjected to chow diet (open circles, female, n = 3; male, n = 4). The two male mice with RYGB not resisting weight gain are indicated by the gray and black dots. Scale bar in A is 250 µm. Data are shown as individual data points overlaid on a box showing mean ± SEM. Groups that do not share the same letters are significantly different from each other (p < 0.05, pairwise t-tests with Benjamini-Hochberg correction, FDR = 0.05).

In both female and male mice liver fat content as visualized with oil-red-O staining was greatly increased in both obese groups (sham and non-surgical high-fat fed controls) compared to non-surgical chow mice (Fig. 8a,b). Importantly, there was only very weak oil-red-O staining in both female and male mice with RYGB. However, while absolute liver weight was more than 3-fold higher in the two male obese groups there were no significant differences in absolute liver weight in females (Fig. 8d). Liver weight normalized to total body weight was significantly higher in female, but lower in male RYGB mice, compared to their respective sham-operated controls (Fig. 8e).

The two male mice with early RYGB that gained significant body weight on the high-fat diet were still somewhat protected from developing hepatosteatosis. In the mouse that gained as much weight as the average sham mouse, oil-red-O staining was less intense (Fig. 8c, upper panel), and liver weight was only slightly elevated (Fig. 8d, black dot). In the male mouse with RYGB that gained about half as much body weight as the shams, there was hardly any hepatic fat accumulation (Fig. 8c, lower panel) and liver weight was in the normal range (Fig. 8d).

### Despite prevention of metabolic impairments, RYGB reduces bone density in both female and male mice

Femur bone density at the end of the observation period was about 35% lower in both female and male mice compared to all other groups (Supplementary Fig. S6). Trabecular density was also significantly reduced, but femur length and cortical thickness were not significantly different between groups.

## Discussion

With obesity and metabolic diseases increasingly afflicting children and adolescents^1,2^, it will be important to investigate long-term effects of therapeutic approaches for prevention and treatment. Behavioral therapies starting in adulthood are the least invasive, but the results so far are relatively disappointing, with up to 80% relapse^8,37^, which has been blamed on the strong counter-regulatory biological responses evoked by calorie restriction^38^. However, behavioral therapy at an early age may be more promising and should be rigorously pursued and researched. With pharmacotherapy waiting for the breakthrough that would make it both safe and efficient for lifelong treatment, bariatric surgery is currently the only effective anti-obesity treatment with strong anti-diabetes effects^39^. Here we wanted to test feasibility and efficacy of RYGB performed in lean adolescent mice on the ability to resist diet-induced obesity and impairment of glycemic control later in life.

Our data show that RYGB in adolescent mice is feasible, with negligible complications or mortality and minimal unintended weight loss. None of the 8 female and 13 male mice undergoing RYGB at 5–7 weeks of age had any complications during the immediate postoperative period. All 8 female mice with RYGB were completely healthy to the end of the 62 week postsurgical observation period, which is remarkable considering that for the last 50 weeks they almost exclusively consumed a Western-style high-fat diet rich in saturated fats. Thus, these mice were completely protected from weight gain and metabolic disease for the equivalent of roughly half the typical human lifespan. Importantly, RYGB at an early age did not inhibit normal growth in female mice.

For male mice the outcome was slightly less favorable, but still quite remarkable when compared to sham-operated and diet-exposed non-surgical controls, which all developed severe obesity, insulin resistance, and fatty liver. In fact, 3 out of 8 sham-operated mice died or were euthanized before termination of the study, showing signs of obesity-associated morbidity. RYGB in male mice did inhibit normal growth, with the severity of inhibition depending on the age when surgery was performed. Interestingly, while higher inhibition of growth before exposure to the high-fat diet (surgery at ~5w/~18 g) resulted in complete protection from the diet later in life, less inhibition of growth (surgery at ~6w/~22 g) resulted in incomplete protection, with 3 out of 8 mice developing different degrees of obesity.

Overall, the findings further support the notion that gastric bypass surgery is not indiscriminately lowering body weight but selectively curbs weight and fat mass gain induced by high-fat diet. In the typical “reversal-of-obesity” model in both humans and rodents, surgery-specific effects are superimposed on acute, non-specific effects such as surgical trauma, analgesics, and the severe hypocaloric diet imposed. This makes it difficult to demonstrate the specific effects of the surgery on food intake and body weight. In the present “prevention-of-obesity” model, the nonspecific effects of surgery are long gone when diet exposure takes place, allowing assessment of the specific effects of surgery on eating patterns and food choice when exposed to obesogenic diets. This model will thus be important for elucidating the molecular mechanisms underlying these specific effects. By assessing candidate mechanisms both before and after exposure to obesogenic and diabetogenic diets or challenges, the separate contributions of direct and indirect effects of the surgery can be distinguished.

Interestingly, this study also shows that despite the beneficial metabolic effects of RYGB surgery, some of its negative sequelae persist; in particular, we show that RYGB decreased bone mineral density in a body weight-independent manner which confirms pervious observations in RYGB operated rats^35,40^, mice^41,42^, and humans^43^.

How are mice with RYGB able to stay lean facing the temptation to consume a palatable, high energy-dense diet? Can they resist overconsumption or can they increase energy expenditure? Our data do not provide unequivocally conclusive evidence for either one of these mechanisms, because of some inherent limitations of the study. In order to minimize non-specific, experimentally-induced disturbances of body weight curves, we intentionally did not make any measurements of energy expenditure before 6 weeks into high-fat diet exposure, and we limited food intake measurements to two relatively short periods. In addition, we did not measure fecal energy loss, which is important for a complete energetic assessment^44^.

Nevertheless, data show that both female and male mice with RYGB can partially resist overconsuming the high-fat diet. The typical first two day binge above normal consumption is reduced by almost half compared to mice without surgery. It is unlikely that this is a purely restrictive physical effect, as we have shown that rats with RYGB, if adequately stimulated, can double their daily calorie intake on a high-fat diet by increasing meal frequency^45^. However, RYGB mice do not seem to be able to fully resist the milder but sustained hyperphagia that follows the acute response. Both female and male mice with RYGB ingest about 3–4 Kcal or 30–40% more energy compared to age-matched mice on regular chow, with body weights and composition being equal (Fig. 4). Reduced palatability-driven food intake in mice with RYGB was also indicated by the slightly higher proportion of total intake from low-fat regular chow. Previous studies in reversal-of-obesity rodent models and humans have consistently found a shift from high-calorie foods to low-calorie foods after RYGB^24,36,46,47^, and see^48^ for a recent review. It will thus be interesting to further analyze food intake patterns and motivational components just before and during early diet exposure in this prevention-of-obesity model, as the acute non-specific traumatic effects of the surgery are absent.

It is important to note that after RYGB not all of the ingested energy is absorbed, as a significant amount is lost in the feces, particularly on a high-fat diet. While it is a limitation of our study that we did not analyze the daily fecal energy content, others using the identical high-fat diet and a similar RYGB procedure in mice have reported that it is about 1–2 Kcal/day^26,49^. Therefore, the actual energy available for RYGB mice may be slightly but significantly lower compared to sham-operated mice and only marginally higher than in non-surgical mice on regular chow.

Both female and male mice seem to increase energy expenditure compared to age-matched, chow-fed mice, with female mice showing an early increase and male mice a late increase. Increased energy expenditure has been previously reported in several reversal-of-obesity rodent models with RYGB^50,51^, while in humans the literature is less clear (see^48^ for a recent review). However, measuring energy expenditure near thermoneutrality was necessary to more clearly reveal this increased energy expenditure of RYGB mice in our study. There may be a limit of indirect calorimetry to identify biologically important differences in energy expenditure that are also statistically significant. In our hands, changes of less than ~10%, which could still be biologically significant usually are not statistically significant or would require many more animals and metabolic chambers (Supplementary Table 1). Nevertheless, our data show that the increased energy expenditure of female mice with RYGB at 6 weeks after diet start was no longer observed at 31 weeks. In contrast, male mice with RYGB showed only marginally increased energy expenditure at 6 weeks but a robust 38% increase at 29 weeks. Taken together, our data suggest that in order to resist diet-induced obesity, mice with RYGB use a combination of reduced energy intake, and increased energy expenditure. However the potential contribution of differences in fecal energy loss needs further investigation.

What might explain the differences in protection between females and males? First, RYGB was performed at a slightly higher age in the male cohort with the three high-fat diet responders (Fig. 3). When RYGB was performed in males of the same age, none gained significant weight after high-fat diet exposure (Fig. 2). Thus, age at surgery could influence protection – the earlier the surgery, the better protection. However, since we have not performed surgery in slightly older females, this conclusion can only be speculative and requires further analysis. Second, the stronger initial response to high-fat diet in sham-operated and non-surgical males compared to females (as illustrated by the steeper weight gain during the first few weeks) could lead to less effectiveness of prior RYGB. Third, the delayed relative increase of energy expenditure after RYGB in males (Fig. 5B) may have been preventing more complete protection from high-fat diet-induced weight gain.

In conclusion, we demonstrate that RYGB is feasible in adolescent female and male mice, with no or minimal growth retardation, respectively. For the first time we demonstrate that RYGB at an early age completely prevents diet-induced obesity later in life in female mice and prevents obesity in a majority but not all male mice. Along with protection from body weight/fat gain comes protection from impaired glycemic control and hepatic steatosis, with an indication that RYGB renders some degree of protection from hepatic steatosis in a weight-independent manner. This protection is remarkable because during a good portion of their lifespan, the mice consumed almost exclusively a Western-style high-fat diet rich in saturated fats. However, one of the clearly negative outcomes of this bariatric surgery in both rodents and humans, namely loss of bone mineral density, was not prevented in this model. Protection of body weight and metabolic impairments seems to be achieved by a combination of reduced energy intake and increased energy expenditure, although additional studies will be necessary to carry out a full energetics analysis. It will also be important to further analyze the molecular mechanisms leading to these changes in energy intake and expenditure that render protection. On the translational side, our findings may also further encourage RYGB surgery in adolescent humans or even children when there is a clear prospect of developing obesity later in life.

## Supplementary information

Supplementary Information

## Data Availability

The datasets generated during and/or analysed during the current study are available from the corresponding author on reasonable request.
