# Supplementary material for: Gastric bypass surgery in lean adolescent mice prevents diet-induced obesity later in life
Source: Sci Rep. 2019 May 27;9:7881. doi: 10.1038/s41598-019-44344-7 (PMC6536499; doi:10.1038/s41598-019-44344-7)
Supplement: Supplementary file 1 — Supplementary Information [file 41598_2019_44344_MOESM1_ESM.pdf]

Supplementary Material

Gastric bypass surgery in lean adolescent mice prevents diet-induced obesity later in life

[1]Michael B. Mumphrey, [1]Zheng Hao, R. [1]Leigh Townsend, [1]Emily Qualls-Creekmore [1]Sangho Yu, [2]Thomas A. Lutz, [1]Heike Münzberg, [1]Christopher D. Morrison, [1]Hans-Rudolf Berthoud

[1]Neurobiology of Nutrition & Metabolism Department, Pennington Biomedical Research Center, Louisiana State University System, Baton Rouge, LA, USA

[2] Institute of Veterinary Physiology, Vetsuisse Faculty, University of Zürich, Zürich, Switzerland

**Female**	29°C		23°C	
Correction	6wk	31wk	6wk	31wk
ANCOVA (total mass)	**+18.0% ***	-0.6%	**+8.5% ***	+6.5%
No correction	+13.8%	-1.2%	+3.8%	+6.2%
Total mass	**+22.0% ***	+0.4%	+13.0%	+10.6%
Lean mass	+12.5%	-6.8%	+2.5%	+0.3%
**Male**				
Correction	6wk	29wk	6wk	29wk
ANCOVA (total mass)	+5.9%	**+39.2% ***	+3.9%	+6.9%
No correction	+3.5%	**+48.8% ***	+1.6%	+9.4%
Total mass	**+10.6% ***	**+48.7% ***	+9.7%	+9.6%
Lean mass	+1.4%	**+39.6% ***	-0.3%	+2.6%

Supplementary Table 1. Percent difference in energy expenditure of mice with RYGB on high-fat diet relative to mice without surgery on chow diet using different correction methods for body weight, and measured at 2 different temperatures and 2 time points after start of high-fat diet exposure. *p < 0.05 based on pairwise *t*-tests with Benjamini-Hochberg correction, FDR = 0.05.

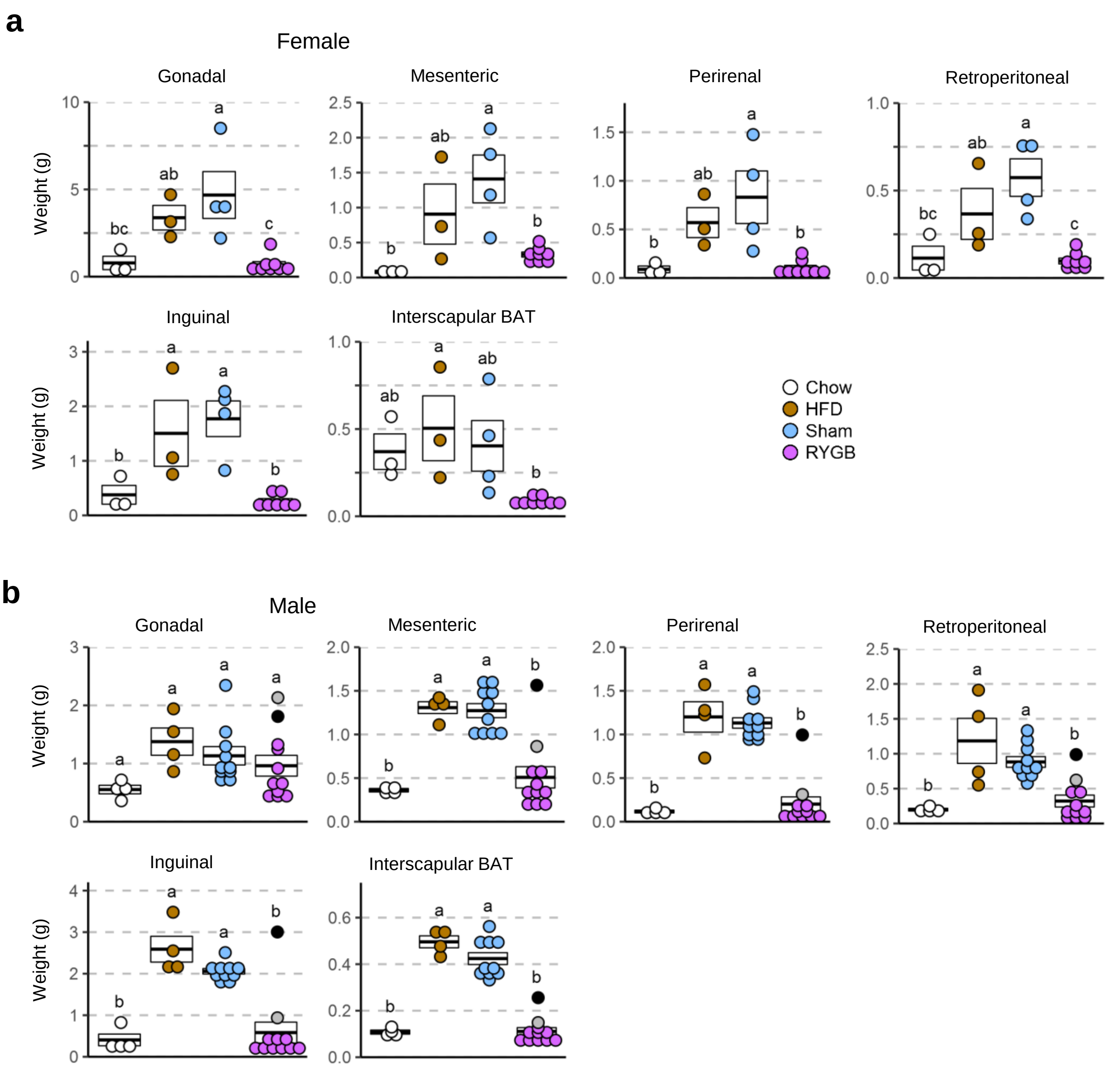

Supplementary Fig. S1. Fat pad weight in female (a) and male (b) mice subjected to RYGB early and exposed to high-fat diet later in life. Weight of fat pads at termination of the study in mice with prior RYGB (purple, Female, n = 8; Male, n = 11), Sham surgery (blue, Female, n = 4; Male, n = 11), no surgery subjected to high-fat diet (brown, Female, n = 3; Male, n = 4), or no surgery subjected to chow diet (open circles, Female, n = 3; Male, n = 4). Note that all data for male mice with RYGB surgery performed at both 5 and 6 weeks were pooled, after determining that there were no significant differences between them. The two male mice with RYGB not resisting weight gain are indicated by the gray and black dots. Data are individual data points overlaid on a box showing mean ± SEM. Groups that do not share the same letters are significantly different from each other ($p < 0.05$, pairwise t-tests with Benjamini-Hochberg correction, FDR = 0.05).

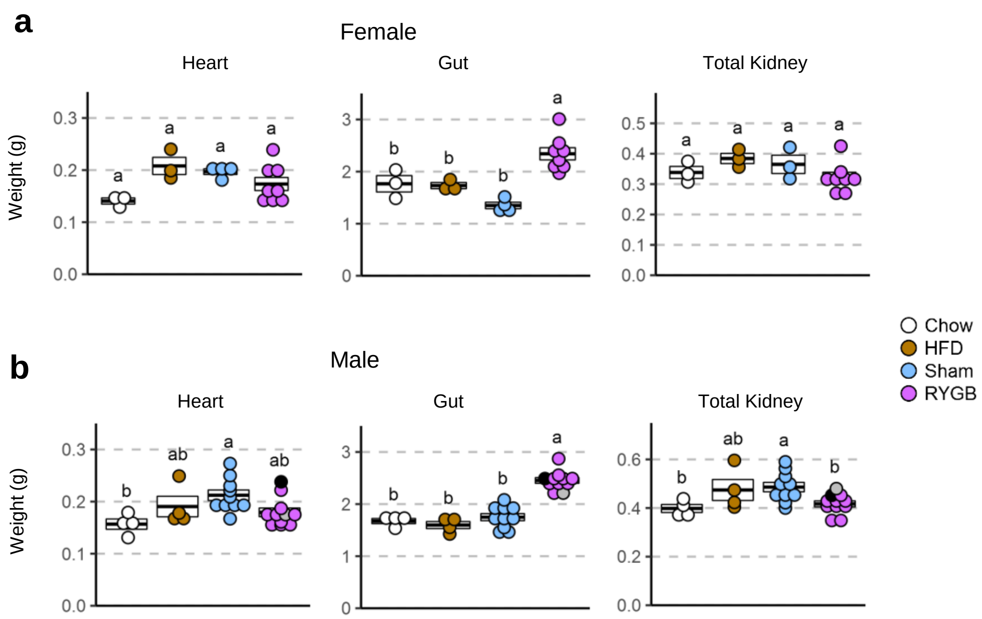

Supplementary Fig. S2. Organ weights in female (a) and male (b) mice subjected to RYGB early and exposed to high-fat diet later in life. Weight of heart, liver, gut, and kidneys at termination of the study in mice with prior RYGB (purple, Female, n = 8; Male, n = 11), Sham surgery (blue, Female, n = 4; Male, n = 11), no surgery subjected to high-fat diet (brown, Female, n = 3; Male, n = 4), or no surgery subjected to chow diet (open circles, Female, n = 3; Male, n = 4). Note that all data for male mice with RYGB surgery performed at both 5 and 6 weeks were pooled after determining that there were no significant differences between them. The two male mice with RYGB not resisting weight gain are indicated by the gray and black dots. Data are individual data points overlaid on a box showing mean ± SEM. Groups that do not share the same letters are significantly different from each other ($p < 0.05$, pairwise t-tests with Benjamini-Hochberg correction, FDR = 0.05).

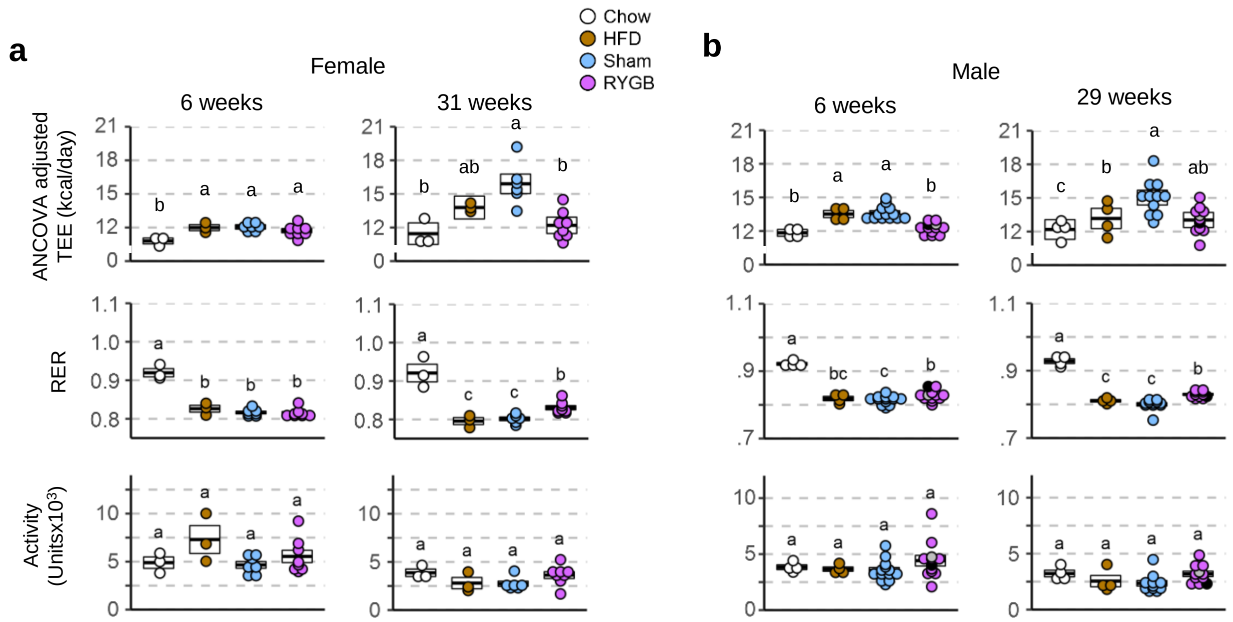

Supplementary Fig. S3. Daily averages of energy expenditure, RER, and activity of female (a) and male (b) mice assessed at two time points after RYGB surgery at room temperature (23^0 C). Metabolic parameters of mice with prior RYGB (purple, Female, n = 8; Male, n = 11-13), sham surgery (blue, Female, n = 6-7; Male, n = 11-13), no surgery subjected to two-choice diet (brown, Female, n = 3; Male, n = 4), or no surgery subjected to chow diet (open circles, Female, n = 3; Male, n = 4), after adaptation, were assessed for 4 days in metabolic chambers, 2 days at 23^0 C and 2 days at 29^0 C (see Fig. 5). Note that all data for male mice with RYGB surgery performed at both 5 and 6 weeks were pooled, after determining that there were no significant differences between them. The two male mice with RYGB not resisting weight gain are indicated by the gray and black dots. Data are individual data points overlaid on a box showing mean ± SEM. Groups that do not share the same letters are significantly different from each other ($p < 0.05$, pairwise t-tests with Benjamini-Hochberg correction, FDR = 0.05).

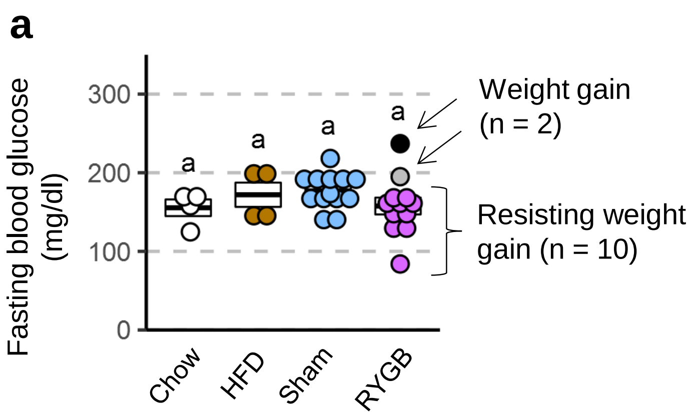

Supplementary Fig. S4. Average fasting blood glucose in male mice. a: Average of fasting blood glucose measured at 13, 25, and 30 weeks after start of diet exposure in male mice with prior RYGB (purple, n = 12), sham surgery (blue, n = 13), no surgery subjected to two-choice diet (brown, n = 4), or no surgery subjected to chow diet (open circles, n = 4). Note the higher average blood glucose of the two outliers gaining body weight. **b:** Same data as in **a** but with outliers omitted. The two male mice with RYGB not resisting weight gain are indicated by the gray and black dots. Data are individual data points overlaid on a box showing mean ± SEM. Groups that do not share the same letters are significantly different from each other ($p < 0.05$, pairwise t-tests with Benjamini-Hochberg correction, FDR = 0.05).

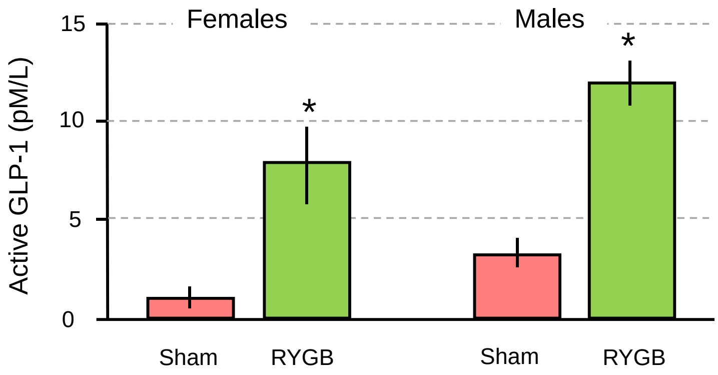

Supplementary Fig. S5. Fasting plasma GLP-1 in female and male mice at termination. Active plasma GLP-1 measured after 3-5 h fasting at termination in females (Sham, n = 4; RYGB, n = 8) and males (Sham, n = 6; RYGB, n = 7). * $p < 0.05$ vs. Sham.

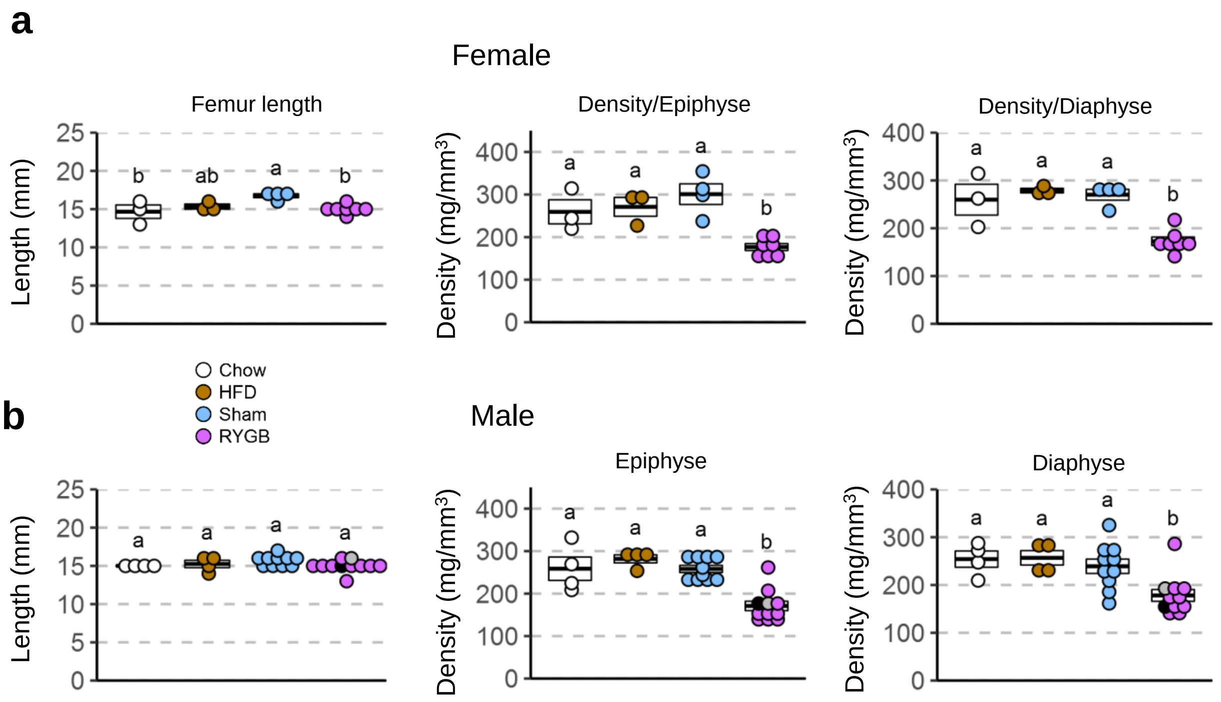

Supplementary Fig. S6. Femur length and density in female (a) and male (b) mice subjected to RYGB early and exposed to high-fat diet later in life. Length, epiphysis density, and diaphysis density of the femur at termination of the study in mice with prior RYGB (purple, Female, n = 8; Male, n = 11), Sham surgery (blue, Female, n = 4; Male, n = 11), no surgery subjected to high-fat diet (brown, Female, n = 3; Male, n = 4), or no surgery subjected to chow diet (open circles, Female, n = 3; Male, n = 4). Note that all data for male mice with RYGB surgery performed at both 5 and 6 weeks were pooled, after determining that there were no significant differences between them. The two male mice with RYGB not resisting weight gain are indicated by the gray and black dots. Data are individual data points overlaid on a box showing mean ± SEM. Groups that do not share the same letters are significantly different from each other ($p < 0.05$, pairwise t-tests with Benjamini-Hochberg correction, FDR = 0.05).
